# *MIR29A* Impedes Metastatic Behaviors in Hepatocellular Carcinoma via Targeting *LOX*, *LOXL2*, and *VEGFA*

**DOI:** 10.3390/ijms22116001

**Published:** 2021-06-01

**Authors:** Ya-Ling Yang, Ming-Chao Tsai, Yen-Hsiang Chang, Chen-Chen Wang, Pei-Yi Chu, Hung-Yu Lin, Ying-Hsien Huang

**Affiliations:** 1 Department of Anesthesiology, Kaohsiung Chang Gung Memorial Hospital and Chang Gung University College of Medicine, Kaohsiung 833, Taiwan; inr453@cgmh.org.tw; 2 Division of Hepato-Gastroenterology, Department of Internal Medicine, Kaohsiung Chang Gung Memorial Hospital and Chang Gung University College of Medicine, Kaohsiung 833, Taiwan; tony0779@gmail.com; 3 Department of Nuclear Medicine, Kaohsiung Chang Gung Memorial Hospital and Chang Gung University College of Medicine, Kaohsiung 833, Taiwan; changyh@cgmh.org.tw; 4 Center for Mitochondrial Research and Medicine, Kaohsiung Chang Gung Memorial Hospital, Kaohsiung 833, Taiwan; 5 Research Assistant Center, Show Chwan Memorial Hospital, Changhua 500, Taiwan; wchen502@gmail.com; 6 Department of Pathology, Show Chwan Memorial Hospital, Changhua 500, Taiwan; 7 School of Medicine, College of Medicine, Fu Jen Catholic University, New Taipei City 242, Taiwan; 8 Department of Health Food, Chung Chou University of Science and Technology, Changhua 510, Taiwan; 9 National Institute of Cancer Research, National Health Research Institutes, Tainan 704, Taiwan; 10 Department of Pediatrics, Kaohsiung Chang Gung Memorial Hospital and Chang Gung University College of Medicine, Kaohsiung 833, Taiwan

**Keywords:** microRNA-29a, hepatocellular carcinoma, metastasis, lysyl oxidase, lysyl oxidase like 2, vascular endothelial growth factor A, diagnosis, prognosis

## Abstract

Primary liver cancer accounts for the third most deadly type of malignant tumor globally, and approximately 80% of the cases are hepatocellular carcinoma (HCC), which highly relies on the activity of hypoxia responsive pathways to bolster its metastatic behaviors. MicroRNA-29a (*MIR29A*) has been shown to exert a hepatoprotective effect on hepatocellular damage and liver fibrosis induced by cholestasis and diet stress, while its clinical and biological role on the activity hypoxia responsive genes including *LOX*, *LOXL2*, and *VEGFA* remains unclear. TCGA datasets were retrieved to confirm the differential expression and prognostic significance of all genes in the HCC and normal tissue. The Gene Expression Omnibus (GEO) dataset was used to corroborate the differential expression and diagnostic value of *MIR29A*. The bioinformatic identification were conducted to examine the interaction of *MIR29A* with *LOX*, *LOXL2*, and *VEGFA*. The suppressive activity of *MIR29A* on *LOX*, *LOXL2*, and *VEGF* was verified by qPCR, immunoblotting, and luciferase. The effect of overexpression of MIR29A-3p mimics in vitro on apoptosis markers (caspase-9, -3, and poly (ADP-ribose) polymerase (PARP)); cell viability and wound healing performance were examined using immunoblot and a WST-1 assay and a wound healing assay, respectively. The HCC tissue presented low expression of *MIR29A*, yet high expression of *LOX*, *LOXL2*, and *VEGFA* as compared to normal control. Serum *MIR29A* of HCC patients showed decreased levels as compared to that of normal control, with an area under curve (AUC) of 0.751 of a receiver operating characteristic (ROC) curve. Low expression of *MIR29A* and high expression of *LOX*, *LOXL2*, and *VEGFA* indicated poor overall survival (OS). *MIR29A*-3p was shown to target the 3′UTR of *LOX*, *LOXL2*, and *VEGFA*. Overexpression of *MIR29A*-3p mimic in HepG2 cells led to downregulated gene and protein expression levels of *LOX*, *LOXL2*, and *VEGFA*, wherein luciferase reporter assay confirmed that *MIR29A*-3p exerts the inhibitory activity via directly binding to the 3′UTR of *LOX* and *VEGFA*. Furthermore, overexpression of *MIR29A*-3p mimic induced the activity of caspase-9 and -3 and PARP, while it inhibited the cell viability and wound healing performance. Collectively, this study provides novel insight into a clinical-applicable panel consisting of *MIR29*, *LOX*, *LOXL2*, and *VEGFA* and demonstrates an anti-HCC effect of *MIR29A* via comprehensively suppressing the expression of *LOX*, *LOXL2*, and *VEGFA*, paving the way to a prospective theragnostic approach for HCC.

## 1. Introduction

Primary liver cancer accounts for the third most deadly type of malignant tumor globally and approximately 80% of the cases are hepatocellular carcinoma (HCC) [1,2]. To date, despite the availability of many different therapeutic interventions, such as surgical resection, liver transplantation, radiofrequency ablation, chemoembolization, and target therapy [3], the prognosis of HCC remains poor, with a five-year overall survival (OS) rate below 20% [1]. One of the key factors is its propensity for metastatic progression and poor response to pharmacological approaches [3]. As such, the identification of promising theragnostic targets to improve the prognosis of the HCC patients is of vital importance.

The lysyl oxidase family members are secreted copper-dependent oxidases, including five paralogues: *LOX*, *LOX*-like 1–4 (*LOXL*1–4), which act to exert catalytic activity to remodel the cross-linking of the extracellular matrix (ECM) of fibrotic liver and that of a corrupted tumor microenvironment (TME) [4]. To date, the roles of *LOX* and *LOXL*2 in the clinical significance and therapeutic implication of HCC are mostly studied as compared to the other family members [5]. The fact that *LOX* and *LOXL*2 serving as critical factors in mediating the formation of a corrupted TME and promoting the progression of metastasis of HCC through activating pathways involved in hypoxia responsive signaling and angiogenesis, and epithelial mesenchymal transition (EMT) has been highlighted [5]. For example, *LOX* acts to mediate angiogenesis via increasing the secreted vascular endothelial growth factor A (*VEGFA*) from HCC cells [6] and to augment metastatic behaviors by activating EMT program [7]. On the other hand, *LOXL*2 was reported to activate the process of angiogenesis through upregulating the HIF-1α/VEGF pathway [8] and promote HCC metastasis to distant organ via an AKT/fibronectin-dependent pathway [9]. Given the biological importance of *LOX*, *LOXL*2, and *VEGFA* in the cancer progression, a variety of targeting drugs has currently been evaluated in clinical trials [5,10,11,12]. Nonetheless, a treatment approach that can comprehensively targeting these factors to render therapeutic potential for the treatment of HCC is yet to be identified.

MicroRNAs (miRNAs), a large family of small non-coding RNAs, was reported to feature diverse and crucial effect on the development and metastasis of HCC by targeting the 3′ untranslational region (3′UTR) of a variety of genes [13]. *MIR29* family members consist of *MIR29A*, *MIR29B*, and *MIR29C*. The UCU sequence of *MIR29A* results in its high stability as compared to the rapid decay of *MIR29B* and *MIR29C* caused by the presence of UUU tri-uracil sequence [14]. The *MIR29A* expression features clinical significance in a variety of disease scenarios, including Alzheimer’s disease [15], Parkinson’s disease [16], ankylosing spondylitis [17], atherosclerosis [18], atrial fibrillation [19], active pulmonary tuberculosis [20], thoracic aneurysms [21], tendon disease [22], diabetes [23], scleroderma [24], cholestatic pediatric liver disease [25], and non-alcoholic fatty liver disease (NAFLD) [26]. Importantly, our previous studies have reported the hepatoprotective role of *MIR29A* in the scenario of cholestasis- or diet-induced steatohepatitis and liver fibrosis [27,28,29,30,31,32,33,34,35,36,37,38], and growing evidence has revealed its regulatory role on HCC metastasis [13,39]. In terms of biological activity, *MIR29A* was reported to directly target *VEGFA* to perturb the wound healing process [40], and to directly target *LOXL2* to impede tumor progression [41]. In this study, we demonstrated that *MIR29A*, along with *LOX, LOXL2*, and *VEGFA*, exhibits diagnostic and prognostic significance and that overexpression of *MIR29A* notably contributes to suppression of cellular metastatic behaviors by comprehensively targeting the 3′UTR of the aforementioned genes, offering novel insights into the *MIR29A*-involved signaling in the development of a practical diagnostic/prognostic panel and a therapeutic strategy.

## 2. Results

### 2.1. The Aberrent Expression and the Prognostic Value of MIR29A, LOX, LOXL2, and VEGFA in HCC Patients

**Figure 1:**
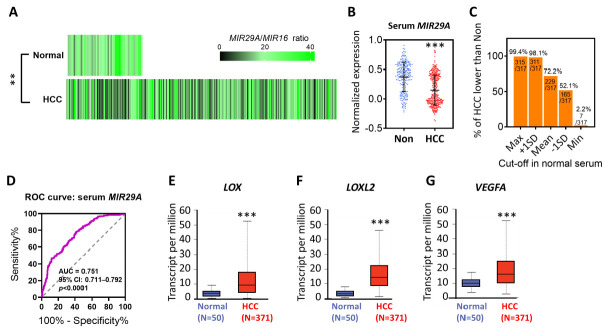
Differential expression analysis of *MIR29A*, *LOX*, *LOXL2*, and *VEGFA* in HCC patients as compared to normal individuals. (**A**) Heatmap of *MIR29A* expression level in normal tissue and HCC, wherein 51 and 375 TCGA datasets were respectively accessed in the OncoMir Cancer Database (OMCD). *MIR16* expression level served as normalization control. ** *p* < 0.01 between normal and HCC group. The raw data of TCGA samples retrieved from OMCD are shown in Supplementary File S1. (**B**) Serum *MIR29A* expression level of HCC patient and non-cancer individuals retrieved from the GSE113740 dataset. *MIR16* expression level served as the normalization control. *** *p* < 0.001 between the non-cancer (Non) and HCC group. (**C**) The number (%) of HCC patients presenting a lower value than normal individuals at the cut-off of maximum, mean + 1SD, mean, mean-1SD, and minimum value of normal serum. (**D**) Receiver operating characteristic (ROC) curves for non-cancer and HCC cohorts based on serum *MIR29A* levels from the GSE113740 dataset. The gene expression level of *LOX* (**E**), *LOXL2* (**F**), and *VEGFA* (**G**) in HCC and normal tissue retrieved from TCGA datasets. ** *p* < 0.01, *** *p* < 0.001 between the normal and HCC group. Panel E, F and G were adapted form UALCAN, which is an open assessed web server.

We firstly examined the *MIR29A* expression levels in HCC and normal tissue by accessing the TCGA datasets in the OncoMir Cancer Database (OMCD). Using *MIR16* as a normalization control as previously described [42], HCC tumor tissue (375 TCGA datasets) presented significantly lower expression levels of *MIR29A* than that of normal tissue (51 TCGA datasets) (*p* < 0.01; Figure 1A). In addition, serum *MIR29A* expression normalized to *MIR16* in HCC patients and non-cancer individuals from the Gene Expression Omnibus (GEO) dataset presented a similar trending (*p* < 0.001; Figure 1B). The number (%) of HCC patients presenting a lower value than normal individuals at the cut-off of maximum, mean + 1SD, mean, mean-1SD, and minimum value of normal serum was 315/317 (99.4%), 311/317 (98.1%), 229/317 (72.2%), 165/317 (52.1), and 7/317 (2.2%), respectively (Figure 1C). We then asked whether *MIR29A* gene alteration in HCC plays a factor in its expression change. In this regard, we surveyed the *MIR29A* data with regard to structural variant, mutation, and copy number alterations (CNA) from TCGA PanCancer Atlas Studies using the cBioPortal web platform, where 10,953 patients/10,967 samples are included as of 26 May 2021. In HCC, *MIR29A* presented a 1.084% alteration frequency in amplification without the presence of deletion and mutation (Supplementary Figure S1), indicating that copy number alteration, deletion, and mutation scarcely contribute to an expression change of *MIR29A*. In addition, *MIR29A* expression between male and female HCC tissue showed no difference (Supplementary Figure S2), suggesting no potential gender bias acting on its expression in HCC tissue. Additionally, we determined the diagnostic value of serum *MIR29A* using a receiver operating characteristic (ROC) curve and noted that *MIR29A* presented good diagnostic accuracy for the discrimination of disease, with an area under curve (AUC) of 0.751 (95% CI: 0.711–0.792, *p* < 0.0001) (Figure 1D). Next, UALCAN web server-accessed TCGA datasets revealed that the mRNA of *LOX*, *LOXL2*, and *VEGFA* presented elevated expression levels in HCC tissue as compared to that in normal tissue (*p* < 0.001; Figure 1E–G).

**Figure 2:**
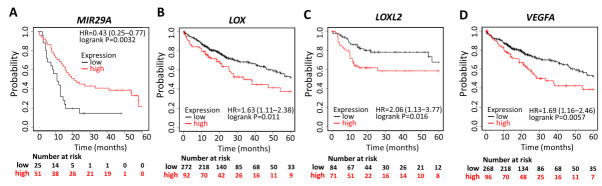
Prognostic value of *MIR29A*, *LOX, LOXL2*, and *VEGFA* in HCC patients. Kaplan–Meier survival analysis representing the probability of the overall survival (OS) in HCC patients based on low vs. high expression of *MIR29A* (**A**), *LOX* (**B**), *LOXL2* (**C**), and *VEGFA* (**D**). HR, hazard ratio.

The correlation between the expression of *MIR29A*, *LOX*, *LOXL2*, and *VEGFA* and corresponding clinical follow-up information was analyzed by Kaplan–Meier curves and the log-rank test. High *MIR29A* expression was found to be associated with increased overall survival (OS) (HR = 0.43; *p* = 0.0032) (Figure 2A), while high expression of *LOX* (HR = 1.63; *p* = 0.011) (Figure 2B), *LOXL2* (HR = 2.06; *p* = 0.016) (Figure 2C), and *VEGFA* (HR = 1.69; *p* = 0.0057) (Figure 2D) was associated with decreased OS. These results indicate that the downregulated *MIR29A* and upregulated *LOX*, *LOXL2*, and *VEGFA* predicts poor prognosis, serve as an independent prognostic panel, and may contribute to HCC progression.

### 2.2. MIR29A Act as A Common Supressor of LOX, LOXL2, and VEGFA

**Figure 3:**
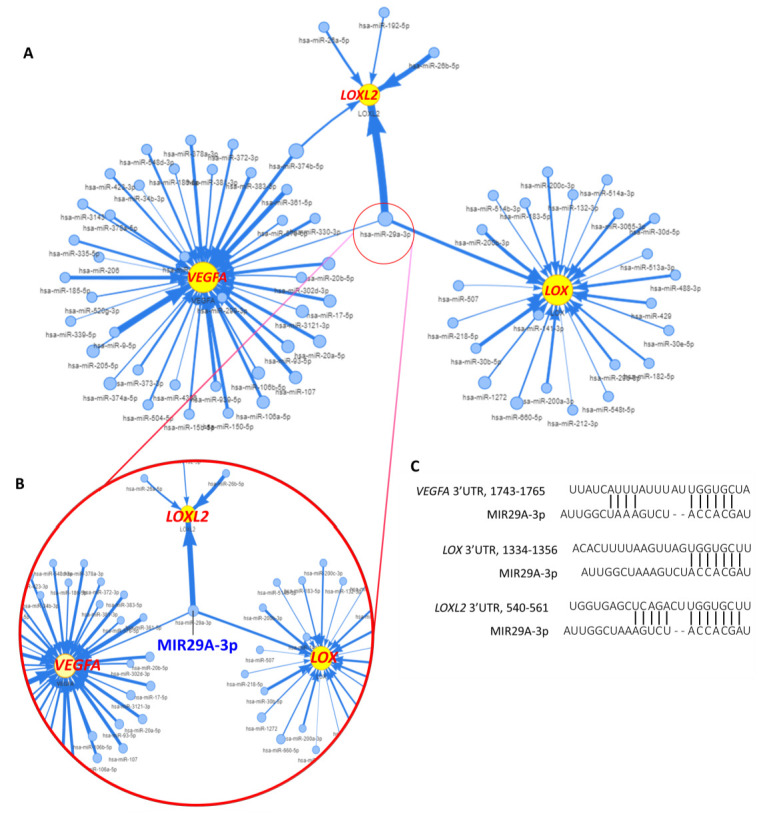
Bioinformatic identification of *MIR29A* as a common suppressor for *LOX, LOXL2*, and *VEGFA* in HCC. (**A**) microRNA-interacting network of *LOX*, *LOXL2*, and *VEGFA* in the TCGA cohort of HCC. (**B**) The enlargement of *MIR29A*-3p-controlled network, revealing targeted genes *LOX*, *LOXL2*, and *VEGFA.* (**C**) *MIR29A*-3p putative binding to the 3′UTR of *LOX*, *LOXL2*, and *VEGFA* were predicted by bioinformatic survey of TargetScan Release 7.2.

**Figure 4:**
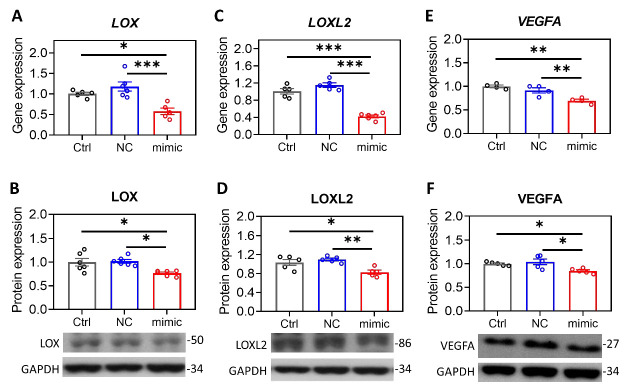
Overexpression of *MIR29A* suppresses the gene and protein expression of *LOX*, *LOXL2*, and *VEGFA*. Human HCC HepG2 cells were treated with transfection reagent as a control (Ctrl), miR negative control sequence (NC), or *MIR29A*-3p mimic (mimic). Then, cell specimens of at least four independent experiments were processed for the detection of gene and protein expression levels of *LOX* (**A**,**B**), *LOXL2* (**C**,**D**), and *VEGFA* (**E**,**F**) by using qPCR and western blot. 18S in qPCR was used for the normalization gene, while GAPDH in western blot served as the loading control. * *p* < 0.05, ** *p* < 0.01, *** *p* < 0.001 between the indicated groups.

**Figure 5:**
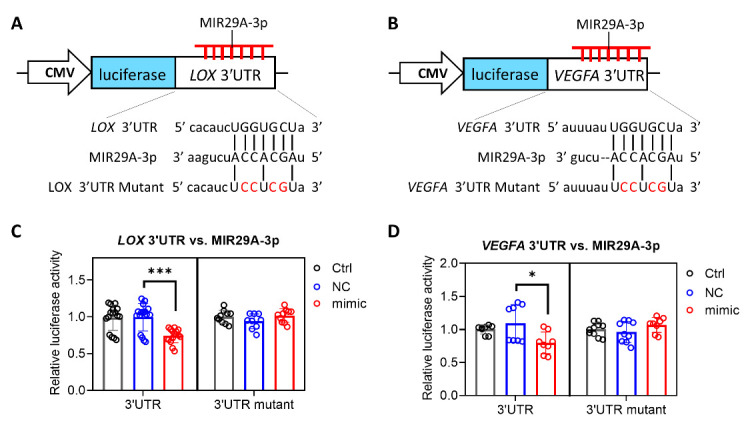
*MIR29A*-3p exerts a suppressive activity by directly targeting the 3′UTR of *LOX* and *VEGFA*. A schematic illustration of the pMIR-REPORTER^TM^ plasmid for the cytomegalovirus (CMV)-driven luciferase reporter assay for *MIR29A*-3p on *LOX* (**A**,**C**) and *VEGFA* (**B**,**D**). Nucleotides in red denotes mismatched mutation as compared to that of wild type. After transfection of the pMIR-REPORTER^TM^ plasmid, HepG2 cells were treated with transfection reagent transfection reagent as a control (Ctrl), miR negative control sequence (NC), or *MIR29A*-3p mimic (mimic), followed by the detection of luciferase activity. * *p* < 0.05, *** *p* < 0.001 between the indicated groups.

The contrary expression pattern between *MIR29A* and the aforementioned mRNAs in HCC tissue prompted us to ask whether *MIR29A* presents a pathway-centric manner on modulating the genes responsible for hypoxic adaptation, matrix remodeling, and angiogenesis. We conducted gene functional enrichment analysis to examine the microRNA-interacting network of *LOX*, *LOXL2*, and *VEGFA* using GSCALite and TargetScan Release 7.2. As shown in Figure 3, *MIR29A*-3p acts as a central hub in regulating *LOX*, *LOXL2*, and *VEGFA* (Figure 3A,B) via putative binding at the 3′UTR (Figure 3C). We then verified the bioinformatic prediction by an in vitro experimental setting, where overexpression of *MIR29A*-3p in human HCC cell line HepG2 significantly inhibited the gene and protein expression of *LOX* (Figure 4A,B), *LOXL2* (Figure 4C,D), and *VEGFA* (Figure 4E,F) (all *p* < 0.05). Additionally, the molecular interaction at posttranscriptional level was corroborated by luciferase reporter assay. As shown in Figure 5A,B, the pMIR-REPORTER^TM^ plasmid comprises a strong protomer CMV to drive the expression of luciferase that is fused with the 3′UTR sequence of *LOX* or *VEGFA* (Figure 5A,B). Binding of *MIR29A*-3p to wild type 3′UTR leads to reduced luciferase signal, whereas the 3′UTR mutant serves as a negative control. Overexpression of *MIR29A*-3p suppressed the reported luciferase activity of wild type but not that of mutant, as compared to the negative control (NC)-treated group (all *p* < 0.05) (Figure 5C,D), revealing that the direct binding to *LOX* 3′UTR and *VEGFA* 3′UTR accounts for the suppression activity of *MIR29A*-3p.

### 2.3. Overexpression of MIR29A Might Induce Apoptosis Signaling and Inhibits Wound Healing Performance and Viability in HCC Cells

**Figure 6:**
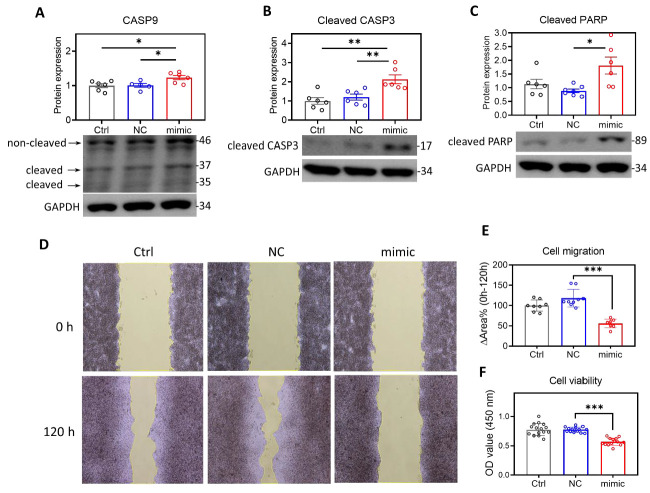
Overexpression of *MIR29A*-3p in HepG2 cells might induce apoptosis signaling and inhibits wound healing performance, and viability. HepG2 cells treated with transfection reagent as a control (Ctrl), MIRs negative sequence (NC), or *MIR29A*-3p mimic (mimic) for 24 h, followed by further assays. The activity of apoptosis signaling was examined by probing the cleaved (37 and 35 kDa)/non-cleaved (46 kDa) caspase-9 (CASP9) (**A**), cleaved caspase-3 (CASP3) (**B**), and cleaved poly (ADP-ribose) polymerase (PARP) (**C**). GAPDH as loading control. The ratio of cleaved/non-cleaved CASP9, and the GAPDH-normalized cleaved CASP3 and cleaved PARP were analyzed for the densitometric quantification. (**D**) Representative image at 0 h and 120 h of wound healing assay. (**E**) Quantification histogram of the changed area percentage (∆Area%) of the scratched ditch determined by the migrated cells. (**F**) Cell viability detected by WST-1-based OD450 signal. * *p* < 0.05, ** *p* < 0.01, *** *p* < 0.001 between indicated groups.

We then evaluated the effect of overexpression of *MIR29A*-3p mimics on the metastatic behaviors of HCC cells. As shown in Figure 6, overexpression of *MIR29A*-3p mimics induced a significant increase in the markers representing activated apoptosis signaling, including the increased ratio of cleaved/non-cleaved caspase-9 (Figure 6A), cleaved caspase-3 (Figure 6B), and cleaved poly (ADP-ribose) polymerase (PARP) (Figure 6C). Additionally, the wound healing assay showed that overexpression of *MIR29A*-3p mimics features a suppressive effect on cellular wound healing performance (Figure 6D,E). WST-1 assay revealed an overexpression of *MIR29A*-3p mimics saps cell viability (Figure 6F). Together, these results indicate that *MIR29A* acts toward the impeded metastatic behaviors of HCC cells.

## 3. Discussion

**Figure 7:**
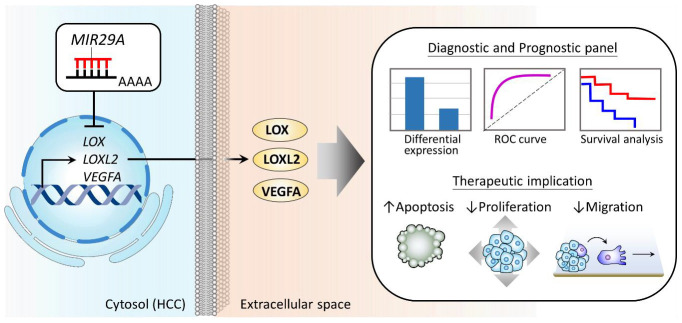
Proposed model illustrating the diagnostic and prognostic value of *MIR29A* and its biological role in suppressing HCC metastatic behaviors. *MIR29A*, *LOX*, *LOXL2*, and *VEGFA* serves as a panel of diagnostic and prognostic factors. Mechanistically, elevated *MIR29A* acts toward the downregulation of *LOX*, *LOXL2*, and *VEGFA*, leading to an impeded metastatic behavior by inducing cell apoptosis and suppressing proliferation and migration.

In this study, we demonstrated that *MIR29A*, *LOX*, *LOXL2*, and *VEGFA* represent a panel of differential biomarkers and independent prognostic factors for HCC patients and that *MIR29A* acts as a common suppressor on these genes to impede the metastatic behaviors of HCC. The proposed model of the clinical value and the mechanism underlying *MIR29A*-centric pathways is illustrated in Figure 7.

There are a number of studies reporting that low expression of *MIR29A* represents a biomarker and a predictor for poor outcome in HCC patients [43,44,45,46], whereas a few reports showed an opposite expression manner [47,48]. In this study, a decreased expression of *MIR29A* is noted in HCC tissue as compared to that of normal tissue and serves as an independent factor for poor OS, in support of the aforementioned reports [43,44,45,46]. Zhu et al. and Xue et al. reported increased serum *MIR29A* as a diagnostic factor of HCC [42,49], while our study demonstrated that serum *MIR29A* expression was in parallel with that of tissue and exhibited a good diagnostic accuracy.

Although *MIR29A*-activated metastasis-suppression signaling pathways involving carcinogenesis, epigenetics, metabolic adaptation, and immunomodulation in HCC have been noted [39], its role in regulating factors modeling the structure of TME was yet to be clarified. In this regard, our study identified *MIR29A* as a common suppressor for *LOX*, *LOXL2*, and *VEGFA*, which notably present a panel of diagnostic and prognostic factors along with MIR29A. As such, this finding provides new insights into clinical decision-making and the development of predictive settings for HCC.

There are increasing reports demonstrating the promising effect of targeting hypoxia-responsive genes, such as *LOX*, *LOXL2*, and *VEGFA*. For instance, a couple of drugs targeting LOX family members are in the early stage of clinical trials [5], including pancreatic and colorectal adenocarcinoma [10,11]. Sorafenib was shown to hit the first breakthrough systemic therapy for treating advanced HCC via disrupting VEGF signaling [50]. Three angiogenesis inhibitor, Trebananib, rebastanib, and MEDI3617, have been evaluated in phase I and II clinical trials [51]. Despite the present lack of HCC trial using approaches targeting the activity of LOX family members, further investigation into broadening the horizons of this issues and the therapeutic strategy widely targeting critical factors are promising. In this study, we demonstrated the biological role of *MIR29A* in comprehensively inhibiting *LOX*, *LOXL2*, and *VEGFA* and its effect on counteracting metastatic behaviors by inducing apoptosis and repressing cell viability and wound healing performance, indicating the potential of *MIR29A* as an adjuvant therapy that may further enhance the treatment effectiveness of the targeting therapy for HCC. Nevertheless, further studies mimicking the physiological circumstance such as in vivo evaluation is warranted to clarify the safety and efficacy in a preclinical setting.

In summary, this study provides novel insight into a clinically applicable panel consisting of *MIR29*, *LOX*, *LOXL2*, and *VEGFA* and demonstrates an anti-HCC effect of *MIR29A* via comprehensively suppressing the expression of *LOX*, *LOXL2*, and *VEGFA*, paving the way to a prospective theragnostic approach for HCC.

## 4. Materials and Methods

### 4.1. Analysis of Gene Differential Expression and Prognostic Significance

The *MIR29A* expression levels in HCC and normal liver tissue were analyzed using the OncoMiR Cancer Database (OMCD) web server, a repository enabling systemic comparative genome analysis of miR expression sequencing data derived from over 10,000 cancer patients with associated clinical information and organ-specific controls present in The Cancer Genome Atlas (TCGA) database [52]. The raw data of TCGA samples retrieved from OMCD are shown in Supplementary File 1. Serum *MIR29A* expression levels in HCC and non-cancer cohorts were analyzed using National Center for Biotechnology Information Gene Expression Omnibus (GEO) database (available online: http://www.ncbi.nlm.nih.gov/geo (accessed on 25 April 2021)) [53], GSE113740. The comparison of the gene expression level in HCC and normal tissue was undertaken in UALCAN web server [54], which facilitates access to publicly available cancer OMICS data, such as TCGA and Clinical Proteomic Tumor Analysis Consortium (CPTAC). *MIR16* served as normalization control as previously described [42].

Kaplan–Meier analysis, which was used to determine the prognostic value of biomarkers, was conducted in the Kaplan-Meier plotter web server (available online: https://kmplot.com/analysis/ (accessed on 25 April 2021)), which includes data sources from GEO, European Genome-phenome Archive (EGA), and TCGA to enable the assessment of the effect of 54 k genes, including mRNA, miR, and protein on survival in 21 cancer types [55]. The prognostic value of miR and mRNA was evaluated by using the panel miRpower for liver cancer [56] and RNA-seq for liver cancer [57] of the KM plotter, respectively.

### 4.2. Bioinformatic Analysis of MIR29A-mRNA Interaction

The microRNA interacting network of *LOX*, *LOXL2*, and *VEGFA2* was retrieved from GSCALite, which is a gene set cancer analysis platform that integrates cancer genomic data from the TCGA [58]. Prediction of *MIR29A*-5p/-3p interaction was conducted using the TargetScan release 7.2 database (available online: http://www.targetscan.org/vert_72/ (accessed on 25 April 2021)) [59].

### 4.3. Cell Culture and Transfection

Human HCC cell line HepG2 were purchased from American Type Tissue Collection (ATCC) and cultured in DMEM medium supplemented with 10% fetal bovine serum (FBS; 10437-028, Thermo Fisher Scientific, Waltham, MA, USA) and 1% penicillin/streptomycin (15240-062, Thermo Fisher Scientific). The oligonucleotides of the *MIR29A* mimics and their corresponding MIR negative control were purchased from GE Healthcare Dharmacon (C-300504-07-0050 and CN-001000-01-50, respectively). The transfection of *MIR29A* mimics and the negative control was carried out with Lipofectamine™ RNAiMAX Transfection Reagent (13778-150, Invitrogen, Carlsbad, CA, USA). Cells were collected 24 h after transfection for further experiments.

### 4.4. Luciferase Reporter Assay

To investigate the molecular basis of *MIR29A*-3p acting on LOX and VEGFA, HepG2 cells were respectively transfected with pMIR-LOX-3′UTR and pMIR-VEGFA-3′UTR followed by transfection of reagent only, MIR negative sequence, or *MIR29A*-3p mimic. Cells transfected with pMIR-LOX-3′UTR mutant and pMIR-VEGFA-3′UTR mutant served as the negative control. Specifically, the sequence of wild type 3′UTR or mutated 3′UTR were cloned into the multiple cloning site after the CMV-driven luciferase (Figure 3B) of the pMIR-REPORTER^TM^ plasmid (AM5795, Thermo Fisher Scientific, Rockford, IL, USA). Then, HepG2 cells were seeded at 1.8 × 10^6^ cells in a 100 mm dish for 18 h. Next, 6 μg reporter plasmids were transfected into a 100 mm dish cultivating HepG2 cells in the presence of Turbofect transfection reagent (R0531, Thermo Fisher Scientific, Rockford, IL, USA) for 20 h. HepG2 were subsequently trypsinized and seeded at 9 × 10^5^ cells in a 60 mm dish with fresh growth medium. Furthermore, 25 nM *MIR29A*-3p mimic (C-300504-07-0050, GE Healthcare Dharmacon, IN, USA), MIR negative control (CN-001000-01-50, GE Healthcare Dharmacon), or no-sequence control (Ctrl) were transfected into cells in the presence of RNAiMAX transfection reagent (#13778-150, Invitrogen, Carlsbad, CA, USA) for 24 h. Finally, cells were lysed for the detection of luciferase signal with Neolite Reporter Gene Assay System (PerkinElmer, Waltham, MA, USA).

### 4.5. Quantitative Real-Time PCR (qPCR)

Total RNA of the cells was extracted by using TRIzol^®^ reagent (15596026, Invitrogen, Carlsbad, CA, USA) and then underwent reverse transcription to yield cDNA with an oligodeoxynucleotide primer (oligo dT15)-based method according to the manufacturer’s protocol (M1701, Promega, Madison, WI, USA). The qPCR reaction was undertaken using 2 × SYBR Green PCR Master Mix (04887352001, Roche Molecular Systems, Inc., Pleasanton, CA, USA) on LightCycler480^®^ (Roche). Each PCR reaction included 0.5 μM forward and reverse primers, 30 ng of cDNA, and 1 × SYBR Green PCR Master Mix in a total reaction volume of 10 μL. For qPCR program, an initial amplification was done with a denaturation step at 95 °C for 10 min, followed by 45 cycles of denaturation at 95 °C for 30 s, primer annealing at 62 °C for 15 s, and primer extension at 72 °C for 25 s, followed by melting curve analysis. The primer sequences for human genes were shown in Table 1.

### 4.6. Western Blotting

The western blotting procedure was compatible with a quantitative approach, as described previously [60]. Briefly, approximately 1 × 10^6^ cells were lysed in protein lysis buffer (17081, iNtRON Biotechnology, Seongnam, Korea), homogenized, and then centrifugated. The resulting supernatant lysates underwent protein quantitation measurement using Bio-RAD protein assay, in accordance with the manufacturer’s protocol (LIT33, Bio-RAD, Hercules, CA, USA). The bovine serum albumin (BSA) was used as a standard to construct a standard curve for the relative quantitation of the proteins in the samples. Next, 40 µg of protein was separated in 8–15%SDS-PAGE, which was then transferred onto PVDF membrane and incubated with primary antibodies at 4 °C overnight. The primary antibodies included LOX (1:1000; NB100-2527, Novus, CO, USA), LOXL2 (1:1000; GTX105085, GeneTex, Irvine, USA), VEGFA (1:1000; ab46154, Abcam, Cambridge, UK), caspase 9 (1:1000; 10380-1-AP, Proteintech, Rosemont, IL, USA), cleaved caspase-3 (1:1000; #9661, Cell Signaling Technology, MA, USA), cleavedPARP (1:1000; #9541, Cell Signaling Technology, MA, USA), and GAPDH (1:100,000; 60004-1-lg, Proteintech). After washing twice with TBST solution, PVDF membrane was incubated with secondary antibodies, such as horseradish peroxidase-coupled anti-rabbit immunoglobulin-G antibodies (1:5000; NEF812001EA, PerkinElmer, Waltham, MA, USA) or HRP anti-mouse immunoglobulin-G antibodies (1:10,000; NEF822001, PerkinElmer) at room temperature for 1 h. The blots were developed with an ECL Western blotting system and exposed them to film (GE28-9068-37, GE Healthcare, Chicago, IL, USA). The signals were quantified by using Quantity One^®^ 1-D analysis software (Bio-Rad Laboratories, Hercules, CA, USA). The accurate quantitative value of the target protein was normalized by its corresponding GAPDH.

### 4.7. Wound Healing Assay

Wound healing assay was employed to detect the wound healing performance of the cells. In each culture-insert, 5 × 10^5^ HepG2 cells were seeded (ibidi culture-insert 2 well, ibidi GmbH, Martinsried, Germany) overnight to allow attachment. Then, the culture-insert was removed, thereby creating a bar of wound. Furthermore, PBS was used to wash out the unattached cells. After the transfection of 25 nM *MIR29A* mimics and their corresponding MIR negative control, the culture plate was photographed to document the width of the wound under a light microscope (50× magnification) at 0 and 120 h. The area of the wound was quantified using imageJ Version 1.53i.

### 4.8. Cell Viability Assay

WST-1 assay was used to determine the cell viability. In each 96-well, 5 × 10^5^ HepG2 cells were seeded at a volume of 100 μL. After attachment, cells were transfected with 25 nM *MIR29A* mimics and their corresponding MIR negative control for 24 h, followed by the addition of 10 μL WST-1 reagent (Roche Diagnostics, Laval, QC, Canada) for another 1 h at 37 °C. The absorbance signal was measured using a microplate reader (Hidex Sense microplate reader, Turku, Finland) at a test wavelength of 450 nm and reference wavelength of 630 nm.

### 4.9. Statistical Analysis

All values are expressed as mean ± standard error (SE). Quantitative data were analyzed using unpaired *t*-test for two-group comparison or one-way analysis of variance for three-or-more-group comparison when appropriate. Least significant difference (LSD) test was used for post-hoc analysis. Two-sided *p*-values less than 0.05 were considered to be statistically significant.

## Figures and Tables

**Table 1:** Sequence of primers pairs.

	Forward Primer	Reverse Primer
LOX	5′-CAGCAGATCCAATGGGAGAAC-3′	5′-GCTGAGGCTGGTACTGTGAG-3′
LOXL2	5′-TGTACCGCCATGACATCGAC-3′	5′-TAGCGGCTCCTGCATTTCAT-3′
VEGFA	5′-AAGGGGCAAAAACGAAAGCG-3′	5′-GCTCCAGGGCATTAGACAGC-3′
18S	5′-GTAAC CCGTT GAACC CCATT-3′	5′-CCATC CAATC GGTAG TAGCG-3′

## Data Availability

Data is contained within the article or Supplementary Material.

## Supplementary Materials

The following are available online at https://www.mdpi.com/article/10.3390/ijms22116001/s1.
