# Supplementary material for: MIR29A Impedes Metastatic Behaviors in Hepatocellular Carcinoma via Targeting LOX, LOXL2, and VEGFA
Source: Int J Mol Sci. 2021 Jun 1;22(11):6001. doi: 10.3390/ijms22116001 (PMC8199573; doi:10.3390/ijms22116001)
Supplement: Supplementary file 1 [file ijms-22-06001-s001.zip › Supplementay Figures.pdf]

Supplementary Information

MIR29A impedes metastatic behaviors in hepatocellular carci-noma via targeting, LOX, LOXL2, and VEGFA

- Supplementary Figure S1…………………..page s1
- Supplementary Figure S2…………………..page s2

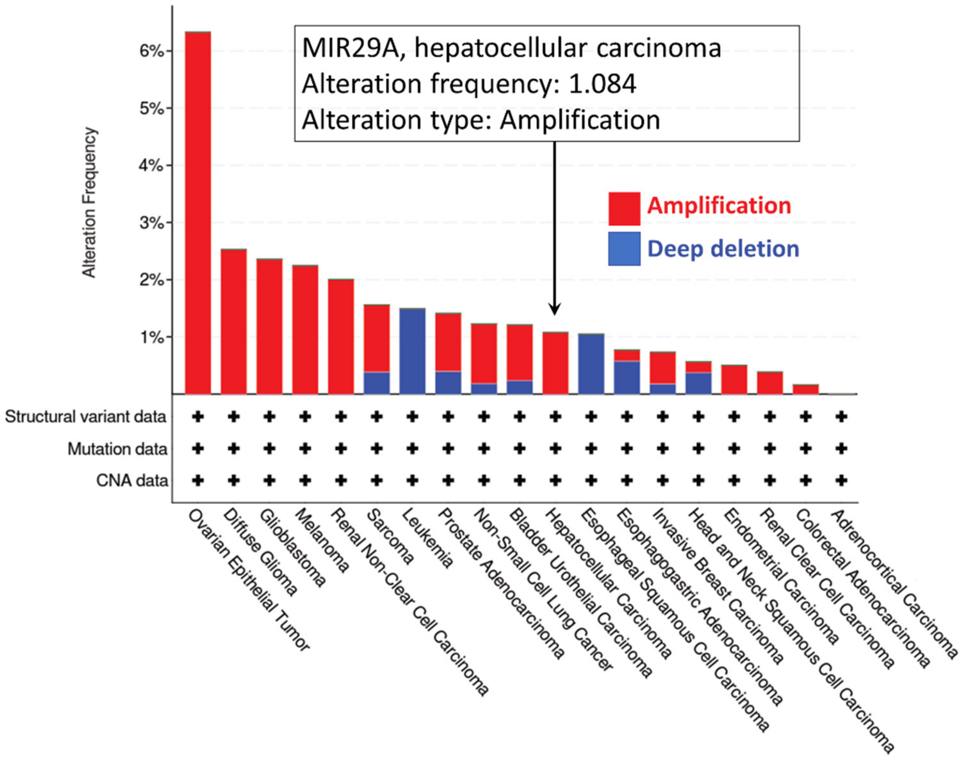

Figure S1. The profile of *MIR29A* gene alteration in various cancer types. Note that *MIR29A* in HCC presented 1.084% of amplification without the presence of deletion and mutation.

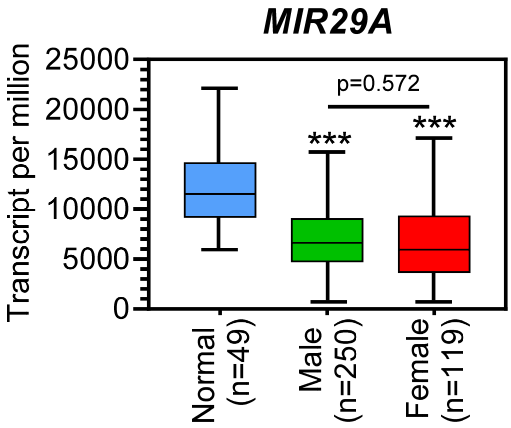

Figure S2. The expression level of *MIR29A* in normal tissue and HCC tumor tissue in male and female. Note that there is no potential gender bias acting on MIR29A expression in HCC (p=0.572 between male and female HCC tissue). ***p<0.001 as compared to normal group.
